# Phytochemical Composition and Biological Activity of Berries and Leaves from Four Romanian Sea Buckthorn (*Hippophae Rhamnoides* L.) Varieties

**DOI:** 10.3390/molecules25051170

**Published:** 2020-03-05

**Authors:** Adriana Criste, Adriana Cristina Urcan, Andrea Bunea, Flavia Roxana Pripon Furtuna, Neli Kinga Olah, Robert H. Madden, Nicolae Corcionivoschi

**Affiliations:** 1 Department of Microbiology and Immunology, Faculty of Animal Science and Biotechnologies, University of Agricultural Sciences and Veterinary Medicine, Cluj-Napoca 400372, Romania; 2 Department of Chemistry and Biochemistry, Faculty of Animal Science and Biotechnologies, University of Agricultural Sciences and Veterinary Medicine, Cluj-Napoca 400372, Romania; andrea.bunea@usamvcluj.ro; 3 SC PlantExtrakt SRL, Rădaia, jud. Cluj 407059, Romania; flavia.plxcercetare@yahoo.com (F.R.P.F.); neliolah@yahoo.com (N.K.O.); 4 Bacteriology Branch, Veterinary Sciences Division, Agri-Food and Biosciences Institute, Belfast BT4 3SD, UK; bob_bt9@yahoo.co.uk (R.H.M.); nicolae.corcionivoschi@afbini.gov.uk (N.C.)

**Keywords:** sea buckthorn, antioxidant activity, antimicrobial activity, carotenoids, flavonoids, phenolic compounds

## Abstract

*Hippophae rhamnoides* L. is an important source of natural antioxidant and antimicrobial agents. Phytochemical compounds, antioxidant and antibacterial properties of berries, and leaf extracts from four Romanian sea buckthorn cultivars were investigated. Large differences in the content of total polyphenols and flavonoids between the varieties were observed. HPLC analysis of the polyphenolic compounds showed greater differences in content in leaves than in berries. This study confirmed that sea buckthorn leaves and berries are a rich source of phenolic compounds, especially quercetin derivatives and hydrocinnamic acid derivatives. Five carotenoid compounds were identified in the berries: lutein, zeaxanthin, β-cryptoxanthin, *cis*-β-carotene, and β-carotene. From the results obtained in this study, it can be stated that the varieties whose berries yielded the highest quantities of polyphenols, flavonoids, and antioxidant activity, can be ranked as follows: SF6 > Golden Abundant > Carmen > Colosal, and for leaf extracts the ranked order is SF6 > Golden Abundant > Colosal > Carmen. A strong correlation between the total flavonoid yield and antioxidant activity (*r* = 0.96), was observed. All extracts showed antibacterial activity against *S. aureus*, *B. cereus*, and *P. aeruginosa*, however extracts from berries were less potent than extracts from leaves.

## 1. Introduction

Over the last few years, there has been an increasing global trend toward the use of natural antioxidants present in fruits and green leafy vegetables because of the fact that consumers are more concerned regarding the safety of using synthetic compounds in convenient food products. The major factors that encourage the use of vegetable sources as antioxidants and antimicrobials are the low cost, plants that contain variable chemical families and amounts of antioxidants with high bioavailability for the human body, and have less adverse effects in the human body compared with synthetic ones.

*Hippophae rhamnoides* L. *ssp. carpatica*, (*Elaeagnaceae* family), commonly known as sea buckthorn (SB), is a thorny, nitrogen-fixing deciduous shrub native to Europe and Asia [1]. There are more than 150 cultivars of sea buckthorn, but new varieties are still being developed [2]. Sea buckthorn berries and leaves are considered to be a rich source of bioactive substances like isoflavones and flavonoids, which have various beneficial effects on health, such as anti-atherogenic, antioxidant, anticancer, and antibacterial effects [3]. The antioxidative properties are attributed to hydrophilic and lipophilic compounds including ascorbic acid, flavonoids, proanthocyanidins, and carotenoids [4,5,6]. In particular, the leaves of sea buckthorn have been reported to contain higher levels of phenolic compounds and antioxidant activities than the berries, and also as having a higher content of nutrients and bioactive compounds such as minerals, vitamins, fatty acids, carotenoids, and phenolic compounds. Antioxidants protect the body from the detrimental effects of free radicals generated as byproducts of normal metabolism and play important roles in preventing pathogenic processes related to cancer and cardiovascular disease, and they can also enhance immune function. In addition to antioxidative roles, phenolic compounds from SB leaves had been reported to have antimicrobial activity against several pathogenic microorganisms. Further, sea buckthorn leaf extracts are reported to have marked antibacterial, antitumoral, anti-inflammatory, and antioxidative activities [7,8,9]. Despite these potential health benefits and their consequent commercial interest, the majority of SB leaves are not used, being classified as agricultural wastes after berry harvesting. Therefore, it may well be worthwhile developing novel products based on the currently unused parts of this plant, and to promote its large-scale utilization.

Factors such as cultivar, harvesting time, and leaf position on the plant, as well as processing technologies, are known to affect significantly the content and composition of phenolic compounds. Antioxidative and antimicrobial activity of sea buckthorn berries and leaf extracts can be related to their bioactive compounds (phenolic, flavonoid, and carotenoid compounds) [10,11]. Up to now, a large number of studies have been carried out on sea buckthorn in different regions of the world, but information about the levels of biologically active compounds and the antioxidant and antimicrobial potential of cultivars grown in Romania is still based on relatively few studies. Thus, the aim of this study was to analyze the bioactive compounds, upon which the bioactive potential of four cultivars of *Hippophae rhamnoides* L. *ssp. Carpatica* is based, in order to provide information allowing the selection of high-activity cultivars for further cultivation. One possible perspective for future use of the obtained data is represented by the possibility to design nutraceutical products for humans and animals based on sea buckthorn. The large volume of “waste” material from sea buckthorn, such as leaves, fruit, pulp, and seed residues from juice and oil extraction, still contain valuable chemical substances that could be developed into a value-added product for animal feed.

## 2. Results and Discussion

Sea buckthorn is reported to be a good source of bioactive compounds due to its content of various phytochemicals, but the specific cultivar is an important factor which affects the content and composition of SB berries and leaves. For these reasons, this study investigated the chemical composition (sugar, protein, and fat) of SB berries and the phytochemical composition (phenolic and flavonoids) of SB berries and leaves from four Romanian cultivars.

### 2.1. Berries Sugar, Protein, and Fat Content

Sugar is an important component of sea buckthorn berries, as it plays a significant role in determining the sweetness of its juice. Typically, only glucose and fructose are reported to be the sugars found in sea buckthorn of all the three major subspecies (*H. rhamnoides ssp. sinensis*, *ssp. rhamnoides*, and *ssp. mongolica*) [12]. One compound detected but often reported as “unknown” was subsequently identified as ethyl β-D-glucopyranoside [13].

For this study, Table 1 presents the biochemical constituents observed in berries from the four Romanian sea buckthorn cultivars studied. Fructose and glucose were the major sugars detected. While the concentrations of fructose in Colosal, Carmen, and Golden Abundant were similar (0.18% ± 0.12%, 0.18% ± 0.25%, and 0.19% ± 0.12%, respectively), it was significantly higher (*p* < 0.05) in the SF6 variety (1.10% ± 0.13%; Table 1).

The glucose content was seen to be higher than that of fructose, and in all cases, it was almost double, but with a similar pattern in each of the cultivars. The glucose concentrations determined were similar to those reported by Yang et al. [12] in all samples of *Hippophae rhamnoides* analyzed, but much lower than the sugar content of *Hippophae sinensis* or *Hippophae mongolica.* The sum of fructose and glucose in the samples analyzed by Yang [14] varied widely from 0.6% in *ssp. rhamnoides* to 24.2% in berries of *ssp. sinensis*, while *ssp*. *mongolica* had a similar level of glucose but less fructose than had *ssp. sinensis*. A glucose/fructose ratio between 1:1 and 1:10 has been reported for sea buckthorn [15], and in this study, the glucose/fructose ratio was at the lower end of those values ranging from 1:1 in the Colosal variety to 1:2.4 in SF6.

Sucrose was only detected, and at a very low level, in the Carmen variety (Table 1), but it was previously reported at trace levels in most of the varieties studied by Yang et al. [12].

This study confirmed that sea buckthorn berries are a rich source of protein and lipids (Table 1). The protein content in SB berries (frequently used as a foodstuff) was observed to be similar in the four varieties analyzed, ranging from 0.72% in Carmen to the highest, 0.86%, in SF 6, and the differences were statistically insignificant.

One special feature of sea buckthorn berry is the high oil content in the soft parts, in addition to the oil found in the seeds [16]. The oil content of the whole berries can vary considerably with the variety and other factors [17]. In our samples, the differences in total fat content are statistically insignificant between Golden Abundant (4.86), SF6 (4.61%), and Colosal (4.21%), but a significantly higher concentration of 5.71% was found in the Carmen variety (Table 1). Previous studies reflect the importance of the specific variety of *Hippophae* spp. in relation to the fat content of fresh berries; the oil level ranges reported range from 1.4% in *ssp. sinensis* from China up to 13.7% in *ssp. turkestanica* from the Western Pamirs [17].

### 2.2. Total and Individual Content of Phenolic Compounds

#### 2.2.1. Total Content of Phenolic Compounds

Total phenolic content (TPC) was determined spectrophotometrically using the Folin–Ciocalteu assay, and was expressed as mg of gallic acid equivalent (GAE)/g. The results, presented in Table 2 below, show that the four varieties of sea buckthorn studied contain notable quantities of phenolic compounds, but that they are significantly higher in the leaves than in the berries.

Statistically significant differences between the total phenolic contents of the varieties of sea buckthorn were recorded (Table 2). Large differences in the content of total polyphenols in berries between varieties were seen, with SF having the highest content (18.66 mg GAE/g), almost double that of Colosal (10.12 ± 0.26 mg GAE/g) or Carmen (10.93 ± 0.38 mg GAE/g).

The TPC of leaves of the different varieties was higher than in berries in all cases, being highest in Golden Abundant (48.12 mg GAE/g) followed by Carmen (42.47 mg GAE/g), Colosal (42.10 mg GAE/g), and SF6 (41.60 mg GAE/g). TPC concentrations ranging from 10.70 to 17.30 (mg GAE/g^−1^) in berries were reported in sea buckthorn by Bittová et al. [18], and the values in Table 2 are in accordance with these. However, Bittová et al. found TPC concentrations in leaves from 72 to 103 mg GAE/g, which, again, indicates the significant differences that exist between varieties of sea buckthorn.

For berries, SF6 had the highest phenolic content (18.66 mg GAE/g fresh weight (FW)), but in contrast, this variety had the lowest phenolic content of all leaf extracts tested (41.60 mg GAE/g FW; Table 2). Overall, the total phenolic content was highest in the leaves of Golden Abundant (*p* < 0.05) in keeping with the finding in this study, and others, that a higher concentration of phenolic compounds was found in the sea buckthorn leaves than in the berries.

The phenolic compounds quantified are considered to be the major determinants for the antioxidant capacity of plants. Therefore, due to their considerable content of phenolics and carotenoids the sea buckthorn plants studied are a promising source of natural antioxidant compounds.

According to Ercisli et al. [19], who studied Turkish sea buckthorn genotypes, their total polyphenolic content varies between 213 and 554 mg GAE 100 g^−1^. In comparison, Korekar et al. [20] analyzed 17 natural populations of sea buckthorn comprising 187 plants from trans-Himalaya and reported that the total phenolic content ranged from 11 to 964 mg GAE/100 g. In contrast, Guo et al. [21], studying the berries of four varieties of sea buckthorn in China, determined values for total phenolic content which averaged only 38.7 ± 1.3 mg GA/g dry weight (DW), emphasizing the need to differentiate between the results obtained from berries and leaves as the former have a lower phenolic content.

Further, Rop et al. [22] demonstrated that the content of polyphenols in sea buckthorn berries is, above all, a matter of cultivars, and it can be very variable. Concerning phenolic content, Rop’s study of six cultivars [22] reported a range of 8.62 g GAE kg^−1^ FM (“Buchlovicky”) to 14.17 g GAE kg^−1^ (“Trofimovskij”), i.e., similar values to those obtained in this study.

Therefore, based on the results of this, and previous studies, sea buckthorn berries have high polyphenol content which varies depending on their genetic background, and the growing and climatic conditions [15,19,23,24]. Hence, although the varieties of sea buckthorn used in this study may differ from those in previous reports, the patterns and levels exhibited by the total phenolic contents of the varieties studied were not dissimilar to the results reported in previous studies.

#### 2.2.2. Flavonoids

The total flavonoid content of the cultivars was determined using an aluminum chloride colorimetric method, and the levels ranged from 9.01 mg quercetin (QE)/g FW in the SF6 berries to 6.57 mg QE/g FW in the Colosal berries (Table 2). Paralleling the total phenolic content, SF6 berries contained the highest total flavonoid concentration and Colosal berries the lowest. Regarding the total flavonoid contents of the leaf extracts, while the levels of flavonoids did not differ markedly, being between 36.58 mg QE/g FW in SF6 leaves and 31.53 mg QE/g FW in Carmen leaves, the former value was significantly greater than in the other three varieties (*p* < 0.05). Moreover, in all leaf extracts, the concentration of flavonoids was significantly higher than in the berry extracts, as was found with the total polyphenols. The results obtained for berries were broadly similar to those determined by Pop et al. [25], who reported that the total flavonols content ranged between 5.63 and 14.37 mg rutin equivalent/g (DW) (“Tiberiu” and “Serpenta” varieties). However. the leaves analyzed in this study were approximately three times richer in flavonols than those examined by Pop et al. [25], who reported values from 8.68 to 13.56 mg rutin equivalent/g DW (“Serbanesti” and “Victoria” varieties).

A recent study of sea buckthorn berries from Poland [26] reported a flavonoid content from 4.63 to 8.93 mg/g DW, i.e., a similar result with those obtained in this study. A study conducted in the Czech Republic investigated the berries of six cultivars of sea buckthorn: “Botanicky” and “Buchlovicky”, which are of Czech origin; “Hergo” and “Leicora”, which are German in origin; and “Ljubitelna” and “Trofimovskij”, which are Russian cultivars [22]. The flavonoids content reported by the study ranged from 4.18 g of rutin kg^−1^ FW to 7.97 g of rutin kg^−1^ FW, while [2] reported that, among cultivars grown in Poland, “Botaniczeskaja-Lubitelskaja” berries had the lowest flavonol concentration (2.12 mg rutin/g FW). Such a great variation of flavonoids indicates that sea buckthorn composition is influenced by many parameters: different subspecies and cultivars, the harvest date, climatic, genetic, and geographic factors, transport, and storage [27], indicating the need for the study reported here, in order to determine which cultivars will produce the greatest yields in a given locality.

#### 2.2.3. Individual Phenolic and Flavonoid Content

HPLC identification of phenolic compounds was made based on their retention time, UV–VIS spectra and mass spectral analysis compared with standards and literature data. Spectral data for all samples were accumulated in the range of 250–360 nm using diode array detection (DAD).

Among the samples analyzed, the HPLC profile of sea buckthorn leaves contained the most complex phenolic fingerprint with 14 different phenolic compounds identified comprising nine flavonoids, cinnamic acid derivatives (four compounds), and gallic acid (one compound). In contrast, berries yielded only 10 different phenolic compounds, but eight flavonoids with only one cinnamic acid derivative (one compound) and gallic acid (one compound). The identification and quantification of phenolic compounds are presented in Table 3.

**Figure 1:**
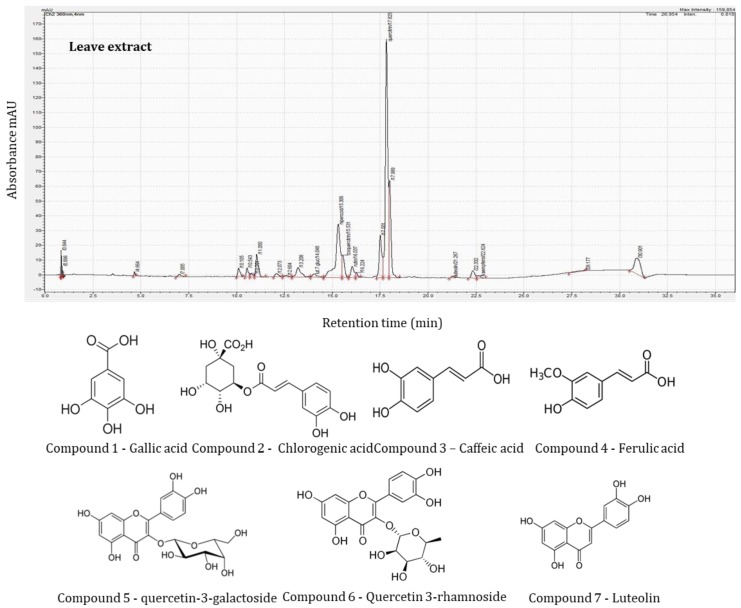
HPLC profile of sea buckthorn leaves extracts.

The HPLC analysis of the polyphenolic compounds of the sea buckthorn showed greater variability of the compounds in leaves than in berries. Thus, in leaf extracts, Golden Abundant (Figure 1) showed a higher quantity of phenols, but a smaller number of phenolic compounds (seven identified compounds), while Carmen and SF6 showed a greater variation in the number of phenolic compounds with 11 and 9 identified compounds, respectively, but with significantly lower levels of them. In the case of berry extracts, this trend was not maintained. Leaf extracts of Colosal yielded the lowest amount of phenols by a significant margin, but the berries gave the highest quantity of phenols.

Significant differences in the content and profile of phenolic compounds have been reported, suggesting that the profile of flavonol glycosides in berries and leaves may be a useful parameter to distinguish between different species of sea buckthorn [26,28].

Generally, the highest content of total polyphenolics was observed in leaf extracts. Quercitrin was the most abundant phenolic compound in all leaf samples, and the significantly highest concentration was found in the sample Golden Abundant at 733 mg/100 g, while the lowest was in Colosal leaf extracts: 152 mg/100 g. Quercitrin, quercetin-3-galactoside, and gallic acid belonged to the most abundant analytes in leaves. Golden Abundant variety was the only one in which caffeic acid was present and Carmen the only variety in which vitexin was identified in the leaves, again emphasizing the variability in composition between varieties.

**Figure 2:**
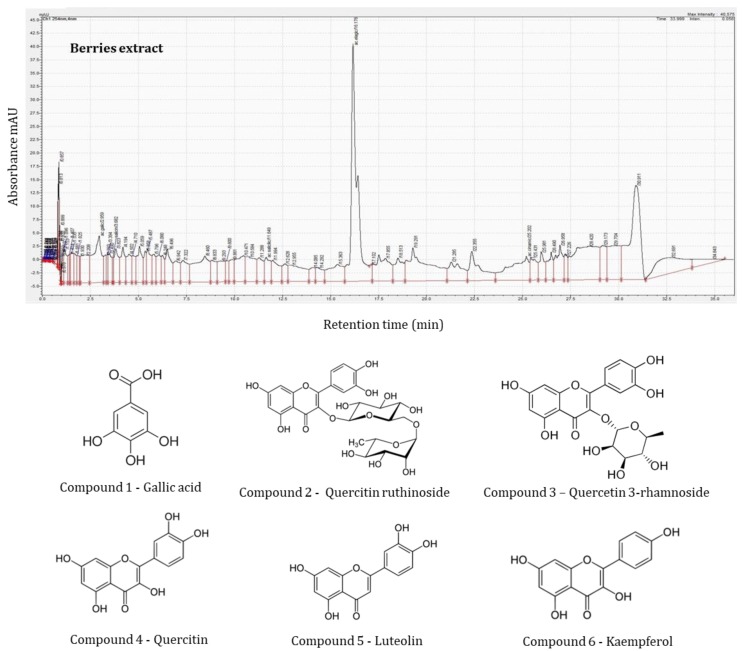
HPLC profile of sea buckthorn berry extracts.

In the case of the sea buckthorn berry extracts the largest concentration of compounds was in Golden Abundant (Figure 2) with 19.37 mg/100 g of gallic acid, followed by Colosal with 18.58 mg/100 g and SF6 with 18.08 mg/100 g (Table 3). While no statistically significant differences (*p* < 0.05) were seen between the gallic acid concentrations in these three varieties, the lowest concentration of gallic acid was in Carmen, yielding only 6.51 mg/100 g and, again, emphasizing the significant differences that can be found between varieties. Gallic acid has been previously reported as the predominant phenolic acid in sea buckthorn berries, at similar levels to those reported in [23]. Besides gallic acid, rutin and quercetin were the dominant phenolic compounds found in the berries. Carmen was the only variety in which vitexin was identified, and luteolin-7-glucoside was present only in Colosal samples.

The most common phenolic compounds identified in this study, in the sea buckthorn berries and leaves, were aglycones, and derivatives of quercetin or cinnamic acid (Table 3).

A reversed-phase high-performance liquid chromatography analysis (RP–HPLC) coupled with diode array detection (DAD) was used to analyze various plant parts (leaves, shoots, berries) of sea buckthorn (*Hippophae rhamnoides* L.) during the annual growth cycle [18]. Catechin, gallic acid, *p*-coumaric acid, caffeic acid, ferulic acid, rutin (quercetin 3-rutinoside), and quercitrin (quercetin 3-rhamnoside) were identified in all leaf samples. As in this study, it was reported that overall the highest content of total polyphenolics was observed in the leaf extracts. Significantly, it was noted that longer maturation of leaf and berries resulted in increased content of quercitrin [18], supporting the contention that plant harvest time can affect its composition. The principal compounds identified in berries were gallic acid, *p*-coumaric acid, ferulic acid, rutin (quercetin 3-rutinoside), and quercitrin (quercetin 3-rhamnoside) [18].

The dominant flavonol identified in this study were also the major compounds identified by Ficzek et al. and Kumar et al. [15,29]. The analyses of Kumar et al. [29] indicated that sea buckthorn leaf extract was rich in rutin, quercetin-3-galactoside, quercetin-3-glucoside, myricetin, quercetin, and kaempferol, which is similar to those identified in the Romanian varieties in this study. The results of this study were also similar to those determined by Ficzek et al. [15] for berries of the German sea buckthorn (*Hippophae rhamnoides* L.) cultivars “Leikora” and “Askola”, and the Siberian cultivar “Orangeveja” with quercetin derivatives, such as the rutin, quercetin glucoside, quercetin dihydrate, quercitrin hydrate, and cinnamic acid being reported.

Based on the results of studies undertaken to date sea buckthorn seems to accumulate flavonoids in a species-specific manner. It has been previously reported that isorhamnetin-3-*O*-rutinoside, isorhamnetin-3-*O*-glucoside, quercetin-3-*O*-rutinoside, quercetin-3-*O*-glucoside (Q3G), isorhamnetin-3-O-glucoside-7-*O*-rhamnoside (I-3-G-7-Rh), isorhamnetin-3-Osophoroside-7-*O*-rhamnoside, kaempferol-3-O-sophoroside-7-*O*-rhamnoside, isorhamnetin, quercetin, kaempferol, gallic acid, ferulic acid, protocatechuic, acid epicathin and catechin were the main compounds in sea buckthorn [2,23,30,31]. Overall, flavonoids are generally recognized as reliable chemotaxonomic markers of plants [32] and, hence, inter- and intra-species differences can be expected.

Teleszko et al. [2] analyzed eight Russian sea buckthorn cultivars (“Aromatnaja”, “Avgustinka”, “Botaniczeskaja”, “Botaniczeskaja Ljubitelskaja”, “Luczistaja”, “Moskwiczanka”, “Podarok Sadu”, and “Porozrachnaja”) and detected 11 flavonols, which were primarily derivatives of isorhamnetin, quercetin. and kaempferol, as found in this study (Table 3).

According to Tkacz et al. [26], isorhamnetin derivatives comprised over 65% of the total flavonols in berries of different cultivars of *Hippophae rhamnoides*, but quercetin and kaempferol derivatives were also determined.

Flavonol glycosides of wild sea buckthorn (*Hippophae rhamnoides ssp. sinensis)* berries from China and cultivated berries (*H. rhamnoides ssp. mongolica*) from Finland and Canada were identified and quantified by Ma et al. [31], who reported 26 flavonol glycosides with isorhamnetin and quercetin being the major aglycones. Again, the content and profile of flavonol glycosides in sea buckthorn berries were highly dependent on the subspecies and cultivars. The study by Pop et al. [25] showed 17 and 19 flavonols in the berries and leaves, respectively, of *H. rhamnoides* L. *subsp. Carpatica* (cv. “Serpenta”, “Serbanesti 4”, “Victoria”, “Sf. Gheorghe”, “Ovidiu”, and “Tiberiu”). Isorhamnetin-3-neohesperidoside, isorhamnetin-3-glucoside, isorhamnetin-3- rhamnosylglucoside, isorhamnetin-3-sophoroside-7-rhamnoside, and free isorhamnetin were predominant in the case of berries. Quercetin-3-hexoside, isorhamnetin-3-hexoside, isoramnetin-3-glucoside-7-rhamnoside, quercetin-3-rutinoside (rutin), quercetin-3-glucoside-7-rhamnoside, and quercetin-3-sophoroside- 7-rhamnoside were predominant in the case of leaves. Yang et al. [33] reported significant quantities of flavonol glycosides in the berries of *ssp. sinensis*, *mongolica*, and *rhamnoides*, with higher levels of isoramnetin-3-glucoside-7-rhamnoside and isoramnetin-3-hexoside, and lower levels for quercetin-3-rutinoside and quercetin-3-glucoside. According to Chen et al. [34], the most effective flavonol glycosides in terms of free radical scavenging activity are isorhamnetin, quercetin, and kaempferol.

Due to significant differences in terms of the flavonol glycosides content of sea buckthorn berries and leaves, these compounds can be considered as biomarkers for the classification or recognition of sea buckthorn belonging to different species and varieties. It is known that the phenolic composition of plant species depends on its chemotype and on various environmental factors [35].

### 2.3. Carotenoid Content

Quantitative HPLC–PAD analysis of saponified carotenoid extract was used to identify carotenoids present in the four sea buckthorn varieties studied, by comparison of the UV–VIS spectra and retention time of sample peaks with those of the standards [5]. Five compounds were identified in the berries: lutein, zeaxanthin, β-cryptoxanthin, *cis*-β-carotene, and β-carotene (Table 4).

As seen with the previous analyses the total carotenoid yield differed significantly between varieties; Carmen had the highest carotenoid content, 35.78 mg/100 g, having more than double the level of the varieties SF6 (10.51 mg/100 g) and Colosal (5.63 mg/100 g). Zeaxanthin and lutein are the predominant carotenoids in the Carmen variety which yielded a higher quantity compared with SF6 or Colosal varieties.

**Figure 3:**
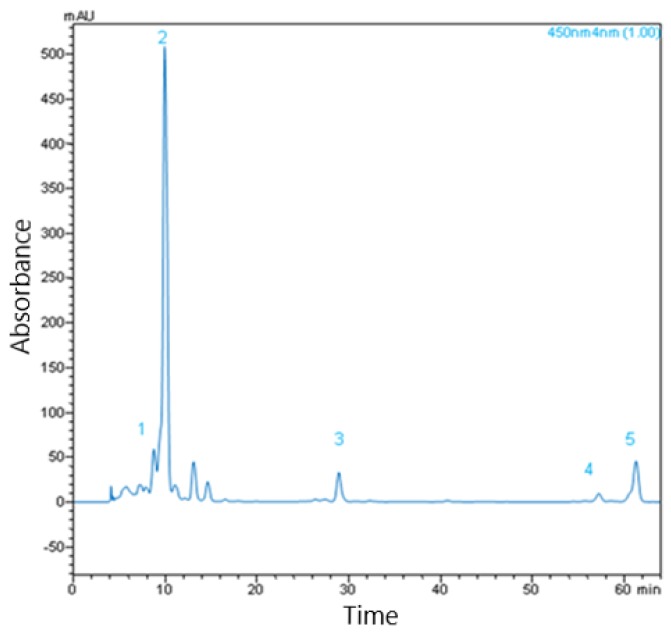
HPLC chromatogram of carotenoids in saponified profile illustrating the separation of carotenoids for the unsaponified extract from Carmen variety sea buckthorn berries. Peak 1—lutein, Peak 2—zeaxanthiyn, Peak 3—β-Cryptoxanthin, Peak 4—*cis*-β-Carotene, Peak 5—β-Carotene

Zeaxanthin (peak 2 in Figure 3), was identified as the major compound in all four Romanian varieties (Table 4), but the concentration differed significantly (*p* < 0.05) between varieties. The lowest amount of this pigment (4.05 g/100 g) was observed in the Colosal sample, while almost double this quantity was found in SF6 (7.62 mg/100 g), and it was even higher in Golden Abundant (16.69 mg/100 g). The highest quantity of zeaxanthin was observed in Carmen variety (27.78 mg/100 g).

In contrast to zeaxanthin, lutein, β-cryptoxanthin, *cis*-β-carotene, and β-carotene were found at significantly (*p* < 0.05) lower levels in all the samples. Paralleling the zeaxanthin content was the lutein and β-carotene content. The quantity of lutein observed was 0.45 mg/100 g in Colosal, 1.02 mg/100 g in SF6, 1.74 mg/100 g in Golden Abundant, respectively, and 4.74 mg/100 g in Carmen, and the quantity of β-carotene observed was 0.17 mg/100 g in Colosal, 0.94 mg/100 g in SF6, 1.94 mg/100 g in Golden Abundant, and almost equal 1.87 mg/100 g in Carmen.

β-Cryptoxanthin and *cis*-β-carotene levels show a different pattern with β-cryptoxanthin being lowest in concentration in Colosal (0.16 ± 0.05 mg/100 g), and higher in the other three varieties; 0.72 mg/100 g in SF6, 1.056 mg/100 g in Golden Abundant, and 1.16 mg/100 g, in Carmen.

The carotenoid found in the lowest concentration was *cis-β*-carotene which varied between 0.21 and 0.80 mg/100 g in all berry extracts, with no significant differences seen between varieties (*p* < 0.05).

The large variations seen in the carotenoid concentration are in agreement with previous studies, which suggest that the carotenoid content varies according to the genetic composition, origin, growing conditions, stage of maturity at harvest, storage conditions, and the methods of analyses. Results reported show concentrations from 27 mg/100 g DW in *Hippophae neurocarpa* from China to 105 mg/100 g in *ssp. turkestanica* from Kyrgyzstan [28,36,37,38].

The values obtained in this study are lower than those reported for other Romanian sea buckthorn varieties [28], where the reported values ranged from 97 mg/100 g in the “Serbănești” variety to 53 mg/100 g in the “Serpenta” variety. This difference is probably due to the fact that in this study saponified samples were analyzed.

Further support for the occurrence of a great variability in the concentrations of carotenoid compounds comes from the analysis of berries from six sea buckthorn cultivars commonly grown in Poland where the total carotenoid concentration found ranged between 47 mg and 509 mg/100 g DW [39].

### 2.4. Antioxidant Potential

Overall, the total polyphenolic and flavonoid content provide general information about the expected antioxidant activity, a property of significant interest from a health point of view, and allow comparison of the antioxidant potential between various samples. Previous research has reported that sea buckthorn berries and leaves have high polyphenol contents, but, as discussed above, the total amount of polyphenols varies greatly depending on many factors, including the cultivar.

Hence, extracts of *Hippophae rhamnoides* L. ssp. *carpatica* were tested to determine their antioxidant activity. Two different methods, 2,2′-azinobis-(3-ethylbenzothiazoline-6-sulphonic acid (ABTS) radical cation quenching and 2-diphenyl-1-picrylhydrazyl (DPPH) radical scavenging, were used to estimate the total antioxidant activity of the samples.

The scavenging activity (H/e transferring ability) of sea buckthorn berries and leaf extracts against 2,2-diphenyl-1-picrylhydrazyl radical (DPPH) was evaluated spectrophotometrically and was expressed as mg Trolox equivalent/g (Table 5).

The antioxidant potential of the varieties, measured by DPPH assay, and found to be between 36.61 and 42.25 µM Trolox/g FW in the case of berries, with the highest antioxidant activity being observed for SF6, and between 123.5 and 138.7 Trolox/g FW in the leaf extracts.

The leaf extracts showed a higher antioxidant capacity, and this is in good agreement with the polyphenolic content. The leaf extract with the highest antioxidant potential was Golden Abundant with the scavenging activity being 138.7 mg Trolox equivalent/g, followed by Colosal, SF6, and Carmen varieties with 133.1, 129.6, and 123.5 mg Trolox equivalent/g, respectively.

These values for scavenging activity were similar to those reported for French sea buckthorn where antioxidant activities determined by DPPH assay ranged from 87.0 to 275.0 mg Trolox equivalent/g dry extract [6]. Similar results were also reported for sea buckthorn cultivars (“Leikora”, “Askola”, “Orangeveja”), grown in Hungary, with the values for antioxidant activity (DPPH method) being between 60.37–79.10 (mg Trolox equivalent/g extract) [15].

The antioxidant potential of the sea buckthorn samples was also measured using the ABTS assay and found to be between 130.6 and 165.2 mg Trolox/g in the leaf extracts, with the highest antioxidant activity again being observed for Golden Abundant. The extracts of sea buckthorn berries analyzed in our study showed a strong capacity of scavenging, with values being between 24.46 and 36.25 mg Trolox equivalent/g, and SF6 had the highest antioxidant activity. Leaf extracts proved to have higher free radical scavenging capacity than berries in accord with the results noted with the DPPH method. A similar observation was made by Kyriakopoulou et al. and Radenkovs et al. [40,41], who reported that sea buckthorn leaves showed higher radical scavenging ability than berries. The total antioxidant activity of different parts of the sea buckthorn plant (leaves, shoots, berries), was determined by using scavenging of the 2,2′-azino-bis(3-ethylbenzothiazoline -6-sulphonate) cation radical (ABTS^+^), and the reported levels were between 37.0 and 50.5 mg GAE/g for leaves and 2.0 and 4.8 mg GAE/g for berries [18].

Based on the data obtained for berries in this study, it can be seen that with regard to the cultivars found to have the highest total phenolics, total flavonoid content, and antioxidant activity, the cultivars are ranked SF6 > Golden Abundant > Carmen > Colosal and considering leaf extracts the order is SF6 > Golden Abundant > Colosal > Carmen. Overall, significant differences can be seen between the results for leaves and berries (*p* < 0.05). A strong correlation between the total flavonoid yield and antioxidant activity, *r* = 0.96, was observed in this study. Overall, the DPPH method yielded slightly higher antioxidant values than did the ABTS method, as can be seen in Table 5.

Bittová et al. [18] investigated the evolution of the antioxidant potential of sea buckthorn (leaves, shoots, and berries) during the annual growth cycle and reported that that sea buckthorn leaf extracts had the highest antioxidant value, while the lowest antioxidant activity was found in extracts from berries. Using the DPPH method, the same author reported leaf extracts as having values between 36.30 and 45.3 mg GAE/g and for berry extracts the values ranged from 1.10 to 2.00 mg GAE/g. The values reported using the ABTS method were slightly higher, however, these values were substantially lower than those reported here (Table 5). As predicted from previous studies, antioxidant activity values followed the same trend as the concentrations of total phenolics, with a strong correlation between the antioxidant parameters being reported [34]. However, some studies could not establish such a strong correlation [19,20,22]. Part of this difference between results given by certain antioxidant assays could be explained by the differing ability of some compounds to react with DPPH and ABTS [20].

Based on the results of this study, and the published literature, it is clear that the antioxidant properties, as in the case of phenolic compounds, vary greatly due the differences found in different varieties of sea buckthorn, and the effects of harvesting time and/or storage conditions used as well as being affected by the use of different methods for estimating scavenging activity.

The use of multiple methods to determine the antioxidant activity helps to better highlight this action. The antioxidant activity of plant extract cannot be evaluated by only a single method due to the complex nature of phytochemicals. In the DPPH assay, the antioxidants such as flavonoids are able to donate hydrogen to reduce the stable radical DPPH to the yellow-colored non-radical diphenyl-picrylhydrazine (DPPH-H). ABTS^+^ radicals are more reactive than DPPH radicals, and the reactions with ABTS^+^ radicals involve a single-electron transfer process and flavonoids, like quercetin derivatives, are able to chelate free radicals immediately by single-electron transfer [42]. Considering the fact that quercitrin was the most abundant compound, and the derivatives of quercetin represented the main flavonoids identified, it is very likely that the antioxidant effect of sea buckthorn samples is attributed to these flavonoids.

It is quite difficult to ascertain which of the compounds most strongly influenced the antioxidant effect, as there is no significant correlation between the results obtained for the concentration of the bioactive compounds and the antioxidant capacity determined for the samples. This study showed not that the extracts that had the greatest abundance of a specific compound had the greater antioxidant effect, but rather that the highest antioxidant effect was obtained with the extracts that had a balanced content of bioactive compounds.

### 2.5. Antimicrobial Activity

In this study, the antimicrobial properties of berry and leaf extracts of four types of *Hippophae rhamnoides L*. ssp. *carpatica* against three bacterial species were determined: *Bacillus cereus, Staphylococcus aureus*, and *Pseudomonas aeruginosa.* The antimicrobial activity of the extracts was assessed by determining the minimum inhibitory concentration (MIC) as indicated in the materials and methods section below.

To determine the impact of sea buckthorn samples on the growth of the different bacterial strains, growth curves were recorded in standard growth medium, a control supplemented with ethanol (100 µL broth + 100 µL ethanol 50%) to mimic the extract solvent, and in standard medium supplemented with sea buckthorn extracts. The results showed that ethanol, at the concentration used, did not influence the growth of the tested bacterial strains, and hence this aspect of the extracts could be ignored.

To determine the minimum inhibitory concentration (MIC) of the plant extracts serial dilutions with 3–100 mg/mL medium were used. MIC was reported as the lowest concentration of the extract causing complete inhibition of the growth of the bacteria. Evaluation of MIC extracts showed some differences in the levels required for inhibition between the bacterial strains studied (Table 6).

All extracts showed some level of antimicrobial activity against the target Gram-positive bacteria: *S. aureus*, *B. cereus*, and Gram-negative bacterium: *P. aeruginosa*, with berry extracts being apparently less potent than leaf extracts. *P. aeruginosa* showed slightly higher sensitivity to the extracts than *S. aureus*, and *B. cereus* showed the highest resistance to the extracts studied. This finding parallels that of Upadhyay et al. [43] who showed the antimicrobial effect of North-West Himalayas sea buckthorn leaves, measured using a disk diffusion method, and a concentration for the aqueous and hydroalcoholic extracts equal to 5 mg/mL. The aqueous extract had the greatest effect against *P. aeruginosa* (18 mm zone of inhibition), while the hydroalcoholic extract gave the greatest inhibition against *B. cereus* (19 mm inhibition). The weakest effects were observed against *E. coli*. Unfortunately, the majority of the literature studies use this disk diffusion method to test antimicrobial activity, due to its simplicity and low cost, but the minimum inhibitory concentration is not determined, leaving a dearth of results to be compared with those found in this study.

The antibacterial activity of sea buckthorn leaf extracts against *S. aureus* (Table 6) was much lower than those obtained by Kumar et al. [29], who determined a MIC of 200 μg/mL with leaf extracts against clinical isolates of *S. aureus* ATCC 12600. Again, this may represent the high variability seen between varieties of sea buckthorn.

The leaf extracts of Golden Abundant and SF6 variety gave higher antimicrobial activity than Carmen and Colosal (Table 6). The higher antimicrobial potential of leaf extracts may be attributed to the levels of polyphenol compounds, especially quercetin derivatives and gallic acid.

Antimicrobial activities of polyphenols can involve various mechanisms, namely destabilization and permeabilization of cytoplasmatic membrane and enzyme inhibition by the oxidized products, possibly through reaction with sulfhydryl groups or through more nonspecific interactions with the proteins, for example, formation of reactive quinones that can react with amino acids and proteins, inhibit the synthesis of nucleic acids of both Gram-negative and Gram-positive bacteria [44]. In particular gallic acid, changed bacterial hydrophobicity [45], while Quercetin may result in bacteriostasis by damaging cell walls and cell membranes [46]. Wang et al. [46] studied the effects of quercetin on cell wall ultrastructure of *E. coli* and *S. aureus*. TEM images revealed that *E. coli* treated with quercetin (50 × MIC) showed the structural integrity and cell membrane damaged, the endochylema density was uneven, cytoplasmic contents leaked, and cell cavitation was evident. In the case of *S. aureus* treated with quercetin (10 × MIC), the extracellular pili of were shed, the cell membrane was damaged, the endochylema density was uneven, and chromatin lysis and nuclear region cavitation were visible. The β galactosidase activity and concentrations of soluble protein in *E. coli* and *S. aureus* significantly increased with increasing quercetin concentrations. The changes in β-galactosidase activity, soluble protein concentrations, and ATP activity of both tested bacteria revealed that the permeability of the cell membrane was affected by quercetin.

Antibacterial properties of crude extracts of sea buckthorn (*Hippophae rhamnoides* L.) pomace, seeds, and leaves from trans-Himalayan Ladakh region, India were tested by Richa Arora et al. [47] against 17 foodborne pathogens. It was reported that leaves yielded the most significant antibacterial activity against 16 tested reference strains out of 17, with *B. cereus* being the most sensitive, with an inhibition zone equal to 17.7 mm, for 50 mg/mL concentration of the extracts. The same study reported that the MIC values for leaf extract, determined by the microdilution broth assay, were 125 μg/mL against *Listeria monocytogenes* ATCC 19111, 250 μg/mL against *Staphylococcus aureus* ATCC 12600, and *Aeromonas hydrophila* and 500 μg/mL against *Salmonella enterica*. Strains of *E. coli* ATCC 8739, *Listeria monocytogenes* ATCC 15313, *Enterococcus faecalis*, and *Klebsiella pneumoniae* were inhibited by 2 to 4 mg/mL concentrations of both aqueous and methanolic extracts. This indicates much higher levels of antimicrobial activity than was found in this study, which may merit further investigation.

Himalayan sea buckthorn leaves have also served as the basis of antimicrobial activity assays against some medically important bacterial species and extracts showed growth-inhibiting effects against *S. aureus*, *E. coli*, *Salmonella typhi*, *Sighela dysenteriae*, and *Streptococcus pneumoniae* at concentrations who ranged between 100 and 500 µ/mL [29]. The leaf extracts had a maximum zone of inhibition (15.2 mm) against *S. dysenteriae* and a minimum zone of inhibition (8.3 mm) for *Streptococcus pneumoniae*. Thus, activity was determined but the assay methodology precludes comparison with the data in this study.

The antimicrobial activities of ethanoic extracts from French *Hippophae rhamnoides* L. leaf, stem, root, and seed at 100 µg/mL were tested against foodborne and clinical microorganisms and the results showed that the maximum inhibition (%) varied according to plant organelle and strain: *S. aureus* (72%) for leaf extract, *Bacillus cereus* (64%) for seed extract, and *Enterococcus durans* (63% and 68%) for root and seed extracts, respectively [6]. *Pseudomonas aeruginosa* was consistently the most resistant to the action of sea buckthorn extracts.

MIC values for extracts of sea buckthorn berries collected from the Lahaul Spiti region (Himachal Pradesh, India) for *B. cereus*, *B. coagulans*, *B. subtilis*, *L. monocytogenes*, and *Y. enterocolitica* were found to be 200, 300, 300, 300, and 350 ppm, respectively, by Negi et al. [48]. Methanolic extract was found to give the highest level of activity, followed by chloroform and acetone extracts.

Positive results were also obtained also by Radenkovs et al. [41] who showed the significant antimicrobial activity of *H. rhamnoides* L. against Gram-positive aerobic or facultative anaerobic and Gram-negative pathogenic bacteria, which included *Bacillus spp*. and the *Enterobacteriaceae* family (*Salmonella spp*., *E. coli*, *Yersinia pestis*, *Klebsiella*, and *Shigella*). This result is also consistent with previous reports that extracts from the other parts of the sea buckthorn plant, such as root, stem, and seeds, possess antimicrobial activities. Jeong et al. [49] showed higher levels of antimicrobial activity studying Korean *H. rhamnoides* (root and stem extracts) when compared with the antimicrobial agents, (+)-catechin, ketoconazol, and mycostantin. That study indicateds that even the plant root and stem contain a variety of compounds contributing to antioxidant and antimicrobial activity and again indicating the potential of this plant in providing benefits to health.

Overall, these findings emphasize the potential for the use of sea buckthorn as a source of natural antioxidants and as the basis for beneficial food supplements.

## 3. Materials and methods

### 3.1. Plant Material

Fresh sea buckthorn berries (SBB) and leaves (SBL), were collected from four varieties (three of them homologated, and referred to as Golden Abundant (SF4), Carmen (SF7), Colosal (SF8), and SF6 (which is not yet homologated). Berries and leaves were harvested in October and were stored in the freezer (−20 °C) until processing.

### 3.2. Chemicals

Standards of sugars, phenolic compounds, and carotenoid compounds were purchased from Sigma-Aldrich Co. and Fluka (Saint Louis, MO, USA) and the rest of the reagents were bought from Merck (Darmstadt, Germany). The samples before analysis were filtered through a 0.45 μm MF-Millipore™ Membrane Filter from Merck (Darmstadt, Germany).

### 3.3. Determination of Sugars

#### 3.3.1. Preparation of Extracts

Samples of sea buckthorn (2.5 g), previously ground using a mortar and a pestle, were homogenized with ultrapure water for 1 h on a multiposition magnetic stirrer (RT series, 10 Carl Roth, Germany). The mixture was transferred to a 50 mL graduated flask containing 12.5 mL methanol of HPLC purity and filled up to the volume with ultrapure water. The solution was filtered through a 0.45 µm Millipore syringe filter, collected in vials, and stored at 4 °C until further analysis.

#### 3.3.2. Determination of Sugars by the HPLC Method

For carbohydrate analysis, the HPLC method developed by Bonta [50] was used, adapted for sea buckthorn berries. A Shimadzu Liquid Chromatograph model SLC-10 Avp with a refractive index detector (HPLC/IR) was used. Chromatographic separation of carbohydrates was performed on an Altima Amino 100 stainless steel column, with a length of 250 mm, diameter of 4.6 mm (Alltech, Nicholasville, KY, USA), and particle size of 5 μm. The temperature inside the column was maintained at 30 °C by means of a CTO-10 AS thermostatic heater. Column pressure was 6.3 MPa. The mobile phase was acetonitrile/water (75:25 *v*/*v*) at a flow rate of 1.3 mL/min and it was filtered through a membrane filter (0.45 µm) before chromatographic analysis. The injection volume was 10 µL. A calibration curve was made for each sugar using standard solutions of different concentrations (0.5–80 mg/mL). The linear regression factor of the calibration curves was higher than 0.9982 for all sugars. Sugars were quantified by comparison of the peak area obtained with those of standard sugars. The results for each sugar were expressed as g/100 g sea buckthorn berries.

### 3.4. Total Protein and Fat Content

The total content of protein and fat was determined using the official methods of the AOAC (Association of Official Analytical Chemists) [51]. The total protein content of sea buckthorn berries was determined using the Kjeldahl method and the total fat content was determined by Soxhlet extraction.

### 3.5. Total and Individual Content of Phenolic Compounds

#### 3.5.1. Preparation of extracts

Each sample of sea buckthorn berries and sea buckthorn leaves (1 g) was individually extracted three times with 5 mL of ethanol:water (50% *v*/*v*) and sonicated (Ultrasonic Cleaner, Sonica, 40 KHz, Milaono, Italy) for 1 h. The resulting mix was centrifuged (15,269× *g*) for 10 min and the supernatants were collected and stored until analysis (4 °C).

#### 3.5.2. Total Phenolic Content

The total phenolic content was determined through the Folin–Ciocalteu method [52]. Briefly, 100 µL Folin–Ciocalteu reagent (0.2 N) was added to 10 µL of extracts and mixed with 80 µL sodium carbonate (Na_2_CO_3_) solution (1 M). After 20 min, the absorbance of the resulting blue color was measured at 765 nm. For the quantification, a calibration curve of gallic acid was prepared with solutions in the range of 0.025–0.15 mg/mL (R^2^ = 0.9992). The results were expressed as mg of GAE (gallic acid equivalent) per g of extract. The assays were run in triplicate.

#### 3.5.3. Flavonoids

Flavonoids were measured by the aluminum chloride colorimetric assay [53] adapted for use on a 96-well microplate reader (Synergy™ HT BioTek Instruments, Winooski, VT, USA), using quercetin as reference standard. An exact volume of 25 µL of appropriately diluted sample was added to 100 µL distilled water and 10 µL of 5% sodium nitrate (NaNO_2_) solution. After 5 min, 15 µL of 10% aluminum chloride (AlCl_3_) was added. At 6 min, 50 µL of 1 M sodium hydroxide was added to the mixture and 50 µL of distilled water. Absorbance of the mixture was determined at 510 nm. For quantification, a calibration curve of quercetin was prepared with solutions in the range of 0.025–0.2 mg/mL (R^2^ = 0.9987). The results were expressed as mg of Qe (quercetin equivalent) per g of extract. The assays were run in triplicate.

#### 3.5.4. Individual Polyphenolic Compounds

Samples (1 g) were extracted with (5 mL) of ethanol: water (50% *v*/*v*) and sonicated for 1 h. The resulting mix was centrifuged (15,269× *g*), for 10 min and the supernatants were collected, microfiltered as above, and used for HPLC/diode array detection (DAD) analysis.

Extracts were analyzed with a Shimadzu Nexera-I HPLC using a silica gel C18 column, Fortis C18, 150 × 2.1 mm × 3 µm system by an acidified water–acetonitrile gradient as detailed below. The solvents were represented by water adjusted to pH 2.5 with 0.1% formic acid (A) and acetonitrile (B). A linear gradient was used, starting with 80% A, decreasing to 60% over the next 5 min, to 40% at the 10 min, to 20% at 15 min, and to 20% over the next 5 min. The concentration of solvent (A) decreased to 10% and was maintained for 5 min, phase A was increased to 20% over the next 5 min, and then increased progressively until it reached 80% at the end of the 40 min analysis. All spectral data were accumulated in the range of 220–400 nm. The linearity of the detector response was checked with the following standards: gallic acid, clorogenic acid, caffeic acid, trans p-cumaric acid, ferulic acid, 7-methoxycumarin, quercetin-3-galactoside, rutin, myricetin, quercitrin, quercetin, luteolin, luteolin-7-glucoside, vitexi, and kaempferol.

### 3.6. Determination of Carotenoid Composition

#### 3.6.1. Preparation of Extracts

The extraction procedure described by Breithaupt et al. [54] was used with slight modifications. Total carotenoids were extracted from sea buckthorn berries (1 g) using methanol:ethylacetate:petroleum ether (1:1:1, *v*/*v*/*v*). Repeated extractions were done under continuous stirring for 4 h in the dark. The combined extracts were filtered and then partitioned successively, using a separation funnel, with water, diethyl ether and saturated NaCl solution. The organic phase was collected and then evaporated using a rotary evaporator. The extract was dissolved in a known volume of ethyl acetate for HPLC–PDA analysis. All extractions were done in triplicate.

#### 3.6.2. Carotenoid Quantification by RP–PAD–HPLC

Carotenoid pigments were identified and quantified using a Shimadzu HPLC system (Shimadzu Corporation, Kyoto, Japan), consisting of an LC-20AT solvent delivery module, a DGU-20A3 degasser, and an SPD-M20A UV–Vis photodiode array detector (PAD). Samples were injected into a Phenomenex C30 column (24 cm × 4.6 mm; particle size: 5 μm). The mobile phase was a mixture of solvent (A) methanol/tert-butyl methyl ether/water (81:15:4, *v*/*v*/*v*) and solvent (B) tert-butyl methyl ether/methanol/water (90:7:3, *v*/*v*/*v*). The gradient began with 1% B at 0 min and increased to 100% B at 160 min according to the method described by Giuffrida et al. [55]. The flow rate was adjusted to 0.8 mL min^−1^. The identification of carotenoids from sea buckthorn samples was carried out by comparison of the UV–VIS spectra and retention time of sample peaks with those of the standard solutions.

### 3.7. Antioxidant Activity

#### 3.7.1. Determination of DPPH Scavenging Activity

The scavenging activity (H/e transferring ability) of sea buckthorn berries and sea buckthorn leaf extracts against 2,2-diphenyl-1-picrylhydrazyl radical (DPPH) was evaluated spectrophotometrically by method of Brand-Williams et al. [56] as described by Velazquez et al. [57], with slight modifications, and adapted for use on a microplate reader. Briefly, an aliquot (40 µL) of appropriately diluted extracts were mixed with 200 µL DPPH solution (0.02 mg/mL). Samples were kept for 15 min at room temperature and then the absorbance was measured at 517 nm. The radical scavenging activity is expressed in milligram equivalent Trolox per gram of sample (mg Trolox equivalent/g).

#### 3.7.2. ABTS Radical Scavenging Activity

The ABTS radical scavenging activity assay was performed according to the method described by Re et al. [58] with some modifications. The ABTS assay is based on the scavenging of the 2,2′-azinobis-(3- ethylbenzothiazoline- 6-sulphonic acid) (ABTS) radical (ABTS*^+^) converting it into a colorless product. The degree of decolorization induced by a compound is related to that induced by Trolox, giving the TEAC value. The ABTS*^+^ cation radical was produced by the reaction between 7 mM ABTS solution and 2.45 mM potassium persulfate solution, stored in the dark at room temperature for 12 h. Before usage, the ABTS*^+^ solution was diluted to an absorbance of 0.700 ± 0.025 at 734 nm with ethanol. For the assay, the resulting solution was mixed with 17 µL of sample. The absorbance was read after exactly 6 min. The standard curve was linear between 0.04 and 0.4 mg Trolox. Results were expressed in milligram of equivalent Trolox per gram of sample (mg Trolox equivalent/g).

### 3.8. Antimicrobial Activity

The antibacterial activity of the SB samples was tested against the Gram-positive bacteria: *Staphylococcus aureus* ATCC 6538P, *Bacillus cereus* ATCC 11778, and the Gram-negative bacterium *Pseudomonas aeruginosa* ATCC 27853. Briefly, the minimum inhibitory concentrations (MIC values) were determined using a 96-well microtiter plate. To prepare standardized inoculums, bacteria were grown in liquid nutrient broth (Merck Darmstadt, Germany, 105443) at 37 °C with constant agitation until the optical density matched a turbidity of a 0.5 McFarland standard. A 100 µL volume of each dilution of the extracts, prepared to concentrations ranged between 3 and 100 mg/mL, were added to 100 µL broth medium and were inoculated with 10 µL of the chosen bacterial species. The plates were incubated under continuous shaking for 24 h at 37 °C in a BioTek Synergy 2 multichannel spectrophotometer (BioTek Instruments, Winooski, VT, USA) and the optical density, OD600 nm, was measured every 15 min. Control samples were incubated in parallel in which the sea buckthorn extracts were not added, and where the microbial cultures hence showed a growth curve according to the specific cultural characteristics of the species. In addition, ethanol control solution, ethanol: water (50% *v*/*v*) was analyzed at the same dilutions as sea buckthorn ethanoic extracts to exclude the possibility that the antimicrobial effect could be attributed to the ethanol used to prepare the extracts. All incubated samples had the same final volume of 200 µL.

### 3.9. Statistical Analysis

All measurements were performed in triplicate and the results were represented as mean ± SEM. Statistical analyses were performed with the GraphPad Prism 8 statistics program. Data statistical analyses were achieved by using one-way ANOVA and Tukey-test. The level of significance was set at *p* < 0.05.

## 4. Conclusions

Differences observed in the biological activities of berry and leaf extracts from four Romanian sea buckthorn (*Hippophae rhamnoides* L.) varieties can be attributed to the inherent combination and complex interaction of bioactive compounds, such as quercetin derivatives, in association with lutolin, kaempferol, gallic acid, trans p-cumaric acid, ferulic acid, lutein, and zeaxanthin. Among the four sea buckthorn cultivars, free radical scavenging capacity measured was positively correlated with the phenolic and flavonoid content. Antibacterial activity can be associated with a high quantity and quality of polyphenols and flavonoids. The presence of quercetin derivatives in berries and leaf extracts found in this study is, therefore, the probable basis of activity against *Bacillus cereus* and *Pseudomonas aeruginosa* strains, while antibacterial activity against *S. aureus* is probably based on vitexin, which is associated with a higher quantity of quercitrin and quercetin-3-galactoside in berries from Carmen cultivars as well as an association with luteolin, luteolin-7-glucoside, and trans p-cumaric acid observed in leaves from Colosal cultivars. Considering all these findings, we can conclude that, some specific varieties of sea buckthorn and different parts of this plant can be used as potential sources of natural antioxidants and antimicrobials, and in order to promote its large-scale utilization, it is necessary to develop new products through the design of innovative functional products for human or animal use.

## Figures and Tables

**Table 1:** Total sugar, protein, and fat content of sea buckthorn berries.

Variety	Fructose (%)	Glucose (%)	Sucrose (%)	Protein (%)	Fat (%)
Golden Abundant	0.19 ± 0.12 ^b^	0.35 ± 0.14 ^a^	nd	0.79 ± 0.12 ^a^	4.86 ± 0.31 ^b^
SF6	1.10 ± 0.13 ^a^	0.46 ± 0.22 ^a^	nd	0.86 ± 0.18 ^a^	4.61 ± 0.29 ^b^
Carmen	0.18 ± 0.25 ^b^	0.24 ± 0.15 ^a^	0.095 ± 0.11	0.72 ± 0.21 ^a^	5.71 ± 0.23 ^a^
Colosal	0.18 ± 0.12 ^b^	0.17 ± 0.14 ^a^	nd	0.75 ± 0.11 ^a^	4.21 ± 0.35 ^b^

nd—not detected; different letters within a column denote significant differences (*p* < 0.05).

**Table 2:** Total content of phenolic compounds in sea buckthorn.

	Sample	Total Polyphenols (mg GAE/g)	Flavonoids (mg Qe/g)
**Berries**	Golden Abundant	14.61± 0.41 ^c^	7.50 ± 0.13 ^b^
	SF6	18.66 ± 0.13 ^a^	9.01 ± 0.23 ^a^
	Carmen	10.93 ± 0.38 ^b^	7.32 ± 0.11 ^b^
	Colosal	10.12 ± 0.26 ^b^	6.57 ± 0.13 ^c^
**Leaves**	Golden Abundant	48.12 ± 0.48 ^a^	33.58 ± 0.46 ^b^
	SF6	41.60 ± 0.62 ^b^	36.58 ± 0.18 ^a^
	Carmen	42.47 ± 0.53 ^b^	31.53 ± 0.63 ^b^
	Colosal	42.10 ± 0.54 ^b^	32.59 ± 0.50 ^b^

Different letters within a column denote significant differences (*p* < 0.05).

**Table 3:** Content of polyphenolic compounds identified in sea buckthorn extracts.

**LEAVES**						
**Compound** **(mg/100 g)**	**RT** **(min)**	**Max Absorption**	**GOLDEN ABUNDANT**	**SF6**	**CARMEN**	**COLOSAL**
Gallic acid	2.89	254 nm	8.10 ± 0.41 ^a^	8.90 ± 0.36 ^a^	0.88 ± 0.45 ^b^	4.69 ± 0.28 ^c^
Chlorogenic acid	6.80	326 nm	3.79 ± 0.19 ^c^	nd	6.24 ± 0.22 ^a^	2.62 ± 0.11 ^b^
Caffeic acid	7.00	nd	2.42 ± 0.16	nd	nd	nd
*Trans* p-cumaric acid	10.02	326 nm	nd	nd	7.32 ± 0.21	nd
ferulic acid	10.75	326 nm	1.37 ± 0.11 ^c^	3.50 ± 0.12 ^a^	0.22 ± 0.05 ^b^	nd
7-methoxycumarin	13.03	337 nm	nd	6.10 ± 0.21 ^c^	21.22 ± 0.65 ^a^	1.51 ± 0.13 ^b^
Quercetin-3-galactoside	15.11	360 nm	91.86 ± 0.87 ^a^	33.92 ± ^d^	80.75 ± 0.82 ^b^	28.37 ± 0.51 ^c^
Rutin	15.88	360 nm	nd	25.74 ± 0.25 ^a^	22.19 ± 0.18 ^b^	5.84 ± 0.11 ^c^
Myricetin	17.37	360 nm	nd	34.13 ± 0.34 ^a^	19.36 ± 0.21 ^b^	nd
Quercitrin	17.70	360 nm	732.8 ± 1.87 ^a^	592.8 ± 1.65 ^b^	330.4 ± 1.27 ^c^	151.5 ± 1.01 ^d^
Quercetin	19.19	360 nm	nd	8.69 ± 0.38	nd	nd
Luteolin	21.26	360 nm	1.73 ± 0.21 ^b^	2.53 ± 0.16 ^a^	nd	nd
Vitexin	22.16	337 nm	nd	nd	9.22 ± 0.11	nd
Kaempferol	22.33	360 nm	nd	nd	3.11 ± 0.18 ^a^	1.50 ± 0.10 ^b^
**Total**			**842.11**	**716.33**	**500.92**	**196.01**
**BERRIES**						
**Compound** **(mg/100 g)**	**RT** **(min)**	**Channel**	**GOLDEN ABUNDANT**	**SF6**	**CARMEN**	**COLOSAL**
Gallic acid	2.89	254 nm	19.37 ± 0.46 ^a^	18.08 ± 0.28 ^a^	6.51 ± 0.12 ^b^	18.58 ± 0.29 ^a^
*Trans* p-cumaric acid	10.01	326 nm	nd	nd	nd	3.17 ± 0.21
Luteolin-7-glucoside	14.06	360 nm	nd	nd	nd	1.87 ± 0.09
Quercetin-3-galactoside	15.11	360 nm	nd	2.35 ± 0.12 ^b^	7.52 ± 0.24 ^a^	nd
Rutin	15.89	360 nm	13.95 ± 0.21 ^a^	11.66 ± 0.15 ^b^	3.31 ± 0.08 ^c^	14.34 ± 0.31 ^a^
Quercitrin	17.71	360 nm	2.95 ± 0.18 ^c^	2.73 ± 0.04 ^c^	7.70 ± 0.19 ^a^	4.48 ± 0.11 ^b^
Quercetin	19.19	360 nm	12.20 ± 0.12 ^a^	12.26 ± 0.34 ^a^	nd	10.64 ± 0.06 ^b^
Luteolin	21.27	360 nm	1.45 ± 0.11 ^b^	1.67 ± 0.15 ^b^	nd	4.01 ± 0.08 ^a^
Vitexin	22.20	360 nm	nd	nd	14.20 ± 0.24	nd
Kaempferol	22.33	360 nm	1.81 ± 0.08 ^a^	1.85 ± 0.11 ^a^	1.33 ± 0.14 ^a^	1.89 ± 0.02 ^a^
**Total**			**51.73**	**35.52**	**40.57**	**58.98**

RT—retention time; nd—not detected; different letters within a row denote significant differences (*p* < 0.05).

**Table 4:** Carotenoid content of saponified sea buckthorn berries.

Compound	Peak	Max Absorption (nm)	RT (min)	GOLDEN ABUNDANT	SF6	CARMEN	COLOSAL
				mg/100 g			
Lutein	1	9.2	421,445,474	1.74 ± 0.04 ^b^	1.02 ± 0.16 ^b^	4.74 ± 0.05 ^a^	0.45 ± 0.07 ^c^
Zeaxanthin	2	10.3	424,449,476	16.69 ± 0.64 ^b^	7.62 ± 0.41 ^c^	27.78 ± 0.55 ^a^	4.05 ± 0332 ^d^
β-Cryptoxanthin	3	38.32	425,449,476	1.05 ± 0.15 ^a,b^	0.72 ± 0.19 ^b,c^	1.16 ± 0.22 ^a^	0.16 ± 0.05 ^c^
*cis*-β-Carotene	4	58.2	340,444,468	0.36 ± 0.08 ^a^	0.21 ± 0.16 ^a^	0.23 ± 0.02 ^a^	0.80 ± 0.20 ^a^
β-Carotene	5	61.6	425,450,477	1.94 ± 0.42 ^a^	0.94 ± 0.36 ^b^	1.87 ± 0.33 ^a^	0.17 ± 0.13 ^b^
**Total**				**21.78**	**10.51**	**35.78**	**5.63**

RT—retention time; different letters within a row denote significant differences (*p* < 0.05).

**Table 5:** Determination of potential for antioxidant activity of extracts of sea buckthorn berries and leaves.

	Sample	DPPH Method (mg Trolox equivalent/g)	TEAC Method (mg Trolox equivalent/g)
**Berries**	Golden Abundant	39.25 ± 0.41 ^b^	32.28 ± 2.35 ^c^
	SF6	42.25 ± 0.23 ^a^	36.25 ± 3.24 ^a^
	Carmen	36.61 ± 0.52 ^b^	24.46 ± 2.78 ^b^
	Colosal	39.25 ± 0.35 ^b^	30.18 ± 2.36 ^c^
**Leaves**	Golden Abundant	138.72 ± 2.76 ^a^	125.25 ± 3.25 ^a^
	SF6	129.59 ± 2.59 ^a,b^	106.28 ± 2.65 ^b^
	Carmen	123.47 ± 1.73 ^a,c^	102.28 ± 2.56 ^b^
	Colosal	133.10 ± 3.21 ^b,c^	120.58 ± 2.85 ^a^

Different letters within a column denote significant differences (*p* < 0.05).

**Table 6:** Antimicrobial activity (MIC) of the extracts of *Hippophae rhamnoides* L. ssp. *carpatica* berries and leaves against four bacterial species.

MIC (mg/mL)								
	Berries				Leaves			
Microorganism	Golden Abundant	SF6	Carmen	Colosal	Golden Abundant	SF6	Carmen	Colosal
*S. aureus*	12.5 ± 1.20 ^c^	12.5 ± 1.64 ^c^	25.0 ± 1.86 ^a^	15.6 ± 1.54 ^b^	6.20 ± 0.54 ^b^	6.20 ± 0.54 ^b^	12.5 ± 1.03 ^c^	25.0 ± 1.44 ^a^
*B. cereus*	25.0 ± 2.35 ^b^	25.0 ± 1.95 ^b^	25.0 ± 2.14 ^b^	31.2 ± 2.32 ^a^	12.5 ± 0.86 ^b^	12.5 ± 1.05 ^b^	12.5 ± 0.92 ^b^	25.0 ± 1.28 ^a^
*P. aeruginosa*	12.5 ± 0.87 ^b^	12.5 ± 1.54 ^b^	12.5 ± 0.98 ^b^	15.6 ± 1.15 ^a^	6.20 ± 0.68 ^b^	6.20 ± 0.72 ^b^	6.20 ± 0.76 ^b^	12.5 ± 1.06 ^a^

MIC—minimum inhibitory concentration. Different letters within a row denote significant differences (*p* < 0.05).
